# A heritable component in sex ratio and caste determination in a *Cardiocondyla *ant

**DOI:** 10.1186/1742-9994-6-27

**Published:** 2009-10-28

**Authors:** Sabine Frohschammer, Jürgen Heinze

**Affiliations:** 1 Biologie I, Universität Regensburg, 93040 Regensburg, Germany

## Abstract

Studies on sex ratios in social insects provide among the most compelling evidence for the importance of kin selection in social evolution. The elegant synthesis of Fisher's sex ratio principle and Hamilton's inclusive fitness theory predicts that colony-level sex ratios vary with the colonies' social and genetic structures. Numerous empirical studies in ants, bees, and wasps have corroborated these predictions. However, the evolutionary optimization of sex ratios requires genetic variation, but one fundamental determinant of sex ratios - the propensity of female larvae to develop into young queens or workers ("queen bias") - is thought to be largely controlled by the environment. Evidence for a genetic influence on sex ratio and queen bias is as yet restricted to a few taxa, in particular hybrids.

Because of the very short lifetime of their queens, ants of the genus *Cardiocondyla *are ideal model systems for the study of complete lifetime reproductive success, queen bias, and sex ratios. We found that lifetime sex ratios of the ant *Cardiocondyla kagutsuchi *have a heritable component. In experimental single-queen colonies, 22 queens from a genetic lineage with a highly female-biased sex ratio produced significantly more female-biased offspring sex ratios than 16 queens from a lineage with a more male-biased sex ratio (median 91.5% vs. 58.5% female sexuals). Sex ratio variation resulted from different likelihood of female larvae developing into sexuals (median 50% vs. 22.6% female sexuals) even when uniformly nursed by workers from another colony.

Consistent differences in lifetime sex ratios and queen bias among queens of *C. kagutsuchi *suggest that heritable, genetic or maternal effects strongly affect caste determination. Such variation might provide the basis for adaptive evolution of queen and worker strategies, though it momentarily constrains the power of workers and queens to optimize caste ratios.

## Background

Studies of intra- and interspecific variation of sex ratios in the social Hymenoptera (bees, wasps, and ants) provide among the most convincing support for the importance of inclusive fitness in evolution. Haplodiploid sex determination in the Hymenoptera (males arise from unfertilized eggs and females from fertilized eggs) results in a closer genetic relatedness of workers to their sexual sisters than to their brothers, while queens are equally related to their male and female offspring [1-3]. Consequently, workers in a colony with a single, singly-mated queen can optimize their inclusive fitness by allocating three times more resources into female than male sexuals, while the queens gain most from an equal sex allocation ratio. When queen numbers and queen mating frequencies vary among colonies relative to the population mean, evolution may lead to split sex ratios: under worker control, individual colonies specialize either for the production of male or female sexuals [4,5].

A large number of empirical studies strongly support the predictions from sex ratio theory (e.g., [6]). In numerous species, workers appear to be capable of biasing sex allocation, i.e., via the selective culling of male larvae or biasing the development of sexuals from female larvae, while in other species a compromise between the interests of queens and workers is achieved (e.g., [7-10]).

Despite this compelling evidence, one fundamental assumption of sex ratio theory has remained largely untested: the optimization of sex ratios in evolution requires heritable genetic variation in this trait. While there is some evidence for genetic influences on sex ratio in solitary Hymenoptera [11,12], little is known on the heritability of sex ratios in social insects. On the contrary, one of the major determinants of sex ratios - the propensity of female larvae to develop into female sexuals rather than workers ("queen bias") - is usually fully controlled by the environment, e.g., colony size, temperature, pheromones, or food quality [13]. A lack of variation in the genetic threshold for caste determining influences might severely constrain the adaptive evolution of sex ratios. The importance of genetic variation on queen bias has rarely been documented, with the best evidence coming from hybrid lineages of harvester ants, where caste is exclusively determined by genotype (e.g., [14-17]), and cross-fostering experiments in the acorn ant *Temnothorax curvispinosus *[18].

Due to local mate competition [19] among wingless males, sex ratios in single-queen colonies of the ant genus *Cardiocondyla *are on average highly female biased but vary strongly between colonies [20,21]. By comparing sex ratios among females belonging to different genetic lineages of *C. kagutsuchi *we studied whether this natural variation might have a heritable basis. Sex ratio studies usually provide only short snapshots of sexual production during one or a few breeding seasons and do not take queen age into account, which might severely affect the likelihood of female larvae to develop into sexuals [22]. In contrast, the short life span of *Cardiocondyla *ant queens (< 1 year; [23]) and the fact that they continuously produce sexuals year-round allowed determining, for the first time, the complete lifetime reproduction of ant queens. *C. kagutsuchi *queens from two different genetic lineages differed significantly in their lifetime sexual production and also in the likelihood of fertilized eggs developing into female sexuals rather than workers. This suggests heritable genetic or maternal effects on sex ratio and queen bias.

## Results

Lifetime sex ratios (female sexuals/all sexuals) in 12 monogynous laboratory colonies set up from female sexuals from seven stock colonies collected on Hawai'i and Kauai ranged from 0.098 to 0.960, with a median of 0.459. To determine the basis of this variation we chose two large stock colonies, A and B, which differed strongly in sexual production and allowed young queens to mate with males from a third stock colony, C, and to found a new single-queen colony assisted by workers from a fourth stock colony, D. Throughout their lives, 39 queens from stock A produced in total 127 males and 1193 virgin queens, while 37 queens from stock B produced 253 males and 314 virgin queens. Numerical sex ratios were significantly different between queens from the two stocks even after all queens that had produced less than five sexuals each during their life were excluded (A: n = 22, virgin queens/all sexuals, median, quartiles: 0.915, 0.778, 0.986; B: n = 16, 0.585, 0.402, 0.735; Mann-Whitney *U*-test, *U *= 98.0, *P *= 0.021).

**Figure 1:**
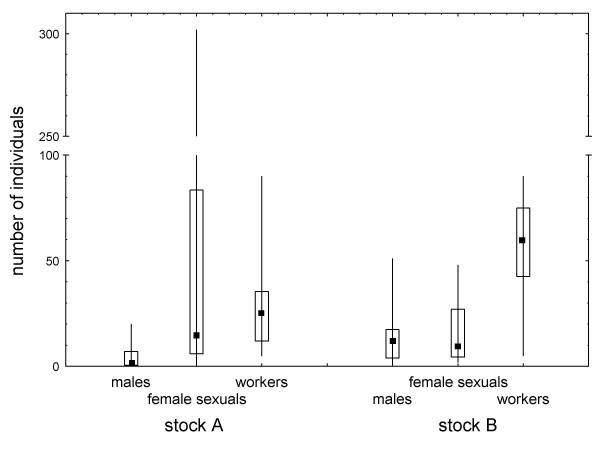
**Number of males, female sexuals, and workers (median, quartiles, range) queens of the ant *Cardiocondyla kagutsuchi *from two different genetic lineages (stock A: 22 queens, stock B: 16 queens) produced during their lives**. Stock A queens produced a significantly more female-biased sex ratio and female larvae with a significantly higher queen bias (female sexuals/all females) than stock B queens.

Queens from the two stocks differed neither in total lifetime offspring number (workers, males, and female sexuals, n_1 _= 22, n_2 _= 16; *U *= 137, *P *= 0.249) nor in total number of diploid offspring (workers and female sexuals, *U *= 144, *P *= 0.344), but in total production of workers (*U *= 65, *P *= 0.001, Fig. 1): queen bias of the female brood was considerably higher in stock A than stock B colonies, both in the total sample (% female sexuals, median, quartiles 50.0, 35.0, 75 vs. 22.6, 10.0, 31.6; *U *= 72, *P *= 0.002) and, at marginal significance, in a comparison between pairs of queens from stock A and stock B that had mated each with exactly the same male (n = 11, Wilcoxon matched pairs test, *T *= 12, *P *= 0.062).

Queens from stock A produced female sexuals significantly earlier (median, quartiles: 42, 35, 50 days vs. 87.5, 59.5, 105.5 days; U = 75.5, p < 0.003) and at much lower worker numbers than colonies of stock B (median, quartiles: 2.5, 0, 5 workers vs. 30, 9.5, 45 workers; U = 75.0, p < 0.003). Furthermore, the time difference between the first appearance of first worker pupae and the first appearance of female sexual pupae was significantly shorter in stock A (median, quartiles: 6.5, 0, 14 days vs. 60, 17.5, 77.5 days; U = 70.0, p < 0.002). First female sexual pupae appeared at the same time as or even before the first worker pupae in 10 of 22 stock A colonies but only 2 of 16 stock B colonies of (Yates corrected χ^2 ^= 4.05, p = 0.044). This all means that, though the first brood was uniformly reared by workers from a different colony D, which originally accompanied each queen, stock A colonies produced relatively more female sexuals than stock B colonies. Indirect sib effects through workers from the different stocks engaging differently in brood care could thus be eliminated.

Our study documents consistent differences in lifetime sex ratios and queen bias between two different lineages of the ant *Cardiocondyla kagutsuchi*. Queens of the two lineages differed significantly in their patterns of reproduction even though they all had mated with males from the same other colony, their first brood was uniformly reared by workers from the same other colony, and their colonies were kept under exactly the same environmental conditions in the laboratory. Sex ratios in *C. kagutsuchi *were different but not because queens limited the number of fertilized eggs and thus forced the workers to rear a male-biased sex ratio, as previously observed in fire ants [24]. Instead, queens of the different lineages produced similar total numbers of diploid offspring, but female larvae differed in the likelihood of developing into queens.

In previous studies, sex ratios and queen bias have usually been estimated only for one or a few subsequent breeding seasons, e.g., for relatively short fractions of the queens' lives. The extremely short life span of *C. kagutsuchi *queens allowed us to determine, for the first time in ants, the exact lifetime reproductive success of each queen. We can therefore exclude a strong influence of queen age on sex ratio and queen bias [22]. Our data instead suggest that caste differentiation and indirectly also sex ratio have a "heritable" component.

Proximately, this can be due to maternal effects, e.g., queens of stock A better provisioning their fertilized eggs (e.g., [22]) or somehow manipulating workers into more provisioning the larvae. Alternatively, stock A larvae might be more sensitive to queen-determining environmental or social influences. A third, indirect factor - workers of the two stocks engage differently in brood care (e.g., [25]) - may also have contributed to the total difference at later stages of brood production. However, queen bias was already different between the two lineages in the very first larvae, which were uniformly reared by workers from another colony. We therefore conclude that eggs or larvae themselves differ in their likelihood of developing into sexuals, either because of direct heritable, genetic or maternal, i.e., queen-derived effects on caste determination.

Genetic caste determination has recently been described from genetically heterogeneous populations of *Pogonomyrmex *harvester ants and *Solenopsis *fire ants, in which fertilized eggs with the genomes of different genetic lineages invariably develop into workers, while genetically homogeneous fertilized eggs invariably develop into queens (e.g., [14-17,26]). Furthermore, genotyping suggested that in colonies of honey bees and leafcutter ants headed by multiply mated queens, female larvae belonging to different patrilines differed in their tendency of developing into female sexuals [27,28]. Our research on *C. kagutsuchi *differs from these studies in several aspects. In contrast to *Pogonomyrmex *and *Solenopsis*, all queens produced both workers and female sexuals from fertilized eggs, although in different proportions. In contrast to honeybees and leaf-cutter ants, the difference in the queen bias of female larvae was expressed not within, but between colonies with a single, singly-mated queen each, which excludes nepotistic brood rearing by workers of a particular genotype (e.g., [29]) as a potential proximate mechanism.

What maintains variation in queen bias in our study population of *C*. *kagutsuchi*? For the evolutionary stability of such a polymorphism, queens, which produce brood with a high queen bias, are expected to have on average the same fitness as queens, which produce brood with a low queen bias. This might be the case if female sexuals from the two lineages differed in their probability of establishing colonies and producing sexuals themselves, e.g., when they followed alternative dispersal and founding strategies. For example, *Leptothorax *sp., an ant with a genetic queen polymorphism, winged queens engage in highly risky dispersal and solitary nest founding. In contrast, wingless queens mate near the maternal nest and found new colonies in a rather safe process called "budding," i.e., assisted by workers from the maternal nest [30]. The efflux of workers from the maternal nest represents a sort of supplementary investment into the female sex and budding in ants appears to be generally associated with a less female-biased numerical sex ratio (e.g., [31]). Wingless queens of *Leptothorax *sp. A indeed produce relatively more workers and fewer female sexuals than winged queens, presumably due to genetic variation in the threshold of queen development [30]. At present little is known about colony founding by *C*. *kagutsuchi *queens, but alternative dispersal strategies like in *Leptothorax *sp. A might be a reasonable explanation for the maintenance of heritable variation in sex ratios and queen bias.

Our study documents heritable variation in queen bias in female larvae of the ant *C. kagutsuchi *and thus in the lifetime production of female sexuals by queens. One might argue that our results reflect idiosyncrasies of particular colonies, as young queens used in this study were taken from two original colonies, which in our study population differed most in their patterns of sexual production. However, exactly such natural variation provides the basis for adaptive evolution of sex and caste ratios required by sex ratio theory. Recent studies have documented genetic variation in several traits of the social phenotype of insect societies that were formerly believed to be mainly due to age or environmental influences, such as division of labour [32], queen size [33], and worker caste polymorphism [34,35]. Our study shows that queen bias and numerical sex ratios may also be influenced by heritable variation. While such genetic variation provides the basis for adaptive evolution, it may momentarily limit the power of workers and queens to optimize caste ratios and might have to be considered as a constraint in studies on sex and reproductive allocation.

## Methods

*C. kagutsuchi *is a complex of morphologically similar species, which are widely distributed throughout Southeast and East Asia [36]. One taxon, which has only wingless males, is a "tramp species" and has been introduced accidentally into many Polynesian islands. On Hawai'i it is one of the very few ants occurring in high densities in primary rain forests with O'hia trees (*Metrosideros *sp.) and Hapu'u tree ferns (*Cibotium*) and also at higher elevations on Mauna Kea volcano [37,38].

Colonies used for this experiment were collected in 2006 near Pu'u o Kila lookout (A, D) and the trailheads of Awa'awapuhi and Nualolo trail (B, C) in Kaua'i, Hawai'i, USA, at an elevation of appr. 1100 m. Colonies were at least 250 m apart and because of mating in the nest and the low dispersal of mated queens can be considered to be, at most, distantly related. We monitored the brood production of the colonies under standard laboratory conditions (e.g., Cremer and Heinze 2002; 17°C/28°C cycles) for approximately one year, during which female sexuals mated with males from their own nests and replaced older queens. We chose two colonies (stocks A and B) with particularly different sex ratios and set up the following experiment: individual female sexuals from stocks A and B were each allowed to mate with a single male from a third colony C and thereafter placed each into a new plastic nest box with brood and 10 workers from a fourth colony D. All foreign brood was removed after the queen had started to lay eggs. The initial addition of workers and brood served to increase colony founding success and to accelerate colony growth. The first brood produced by stock A and stock B queens was thus uniformly reared by workers all from the same stock colony D. This removes the possibility for indirect effects, such as variation in brood care, contributing to the observed differences between lineages. Workers of *Cardiocondyla *do not possess ovaries and therefore did not contribute to the male offspring produced in these colonies.

We originally aimed at pairing each male from colony C both with a queen from stock A and a queen from stock B and to compare brood production between these dependent samples. As several matings failed and a few queens died without producing sufficient numbers of sexuals, only 11 samples were available for a matched pairs test. Numerical sex ratios in single-queen colonies of *Cardiocondyla *have previously been shown to be female-biased because of local mate competition among the wingless fighter males [20,39].

Colonies were kept in incubators as described previously (e.g., [20]) and provisioned with diluted honey and pieces of cockroaches twice per week. The presence of eggs, larvae, and sexual pupae and the number of workers was noted once per week. All sexual pupae were removed. We estimated "worker production" by subtracting the initial workforce, 10, from the maximum number of workers observed in the colony. Queens, which did not produce any female offspring (female sexual or worker) or less than five sexuals were excluded from the analysis.

Data were not normally distributed (Shapiro-Wilks' test, p < 0.05) but variances were homogeneous (Brown-Forsyth test, p > 0.05). We therefore used Mann-Whitney U-tests and Wilcoxon matched pairs tests to compare sex ratios and production data.

## Competing interests

The authors declare that they have no competing interests.

## Authors' contributions

JH collected colonies, and planned the study. SF conducted the research, and both authors analyzed the data and wrote the paper. All authors read and approved the final manuscript.
